# Antimicrobial resistance of bacteraemia in the emergency department of a German university hospital (2013–2018): potential carbapenem-sparing empiric treatment options in light of the new EUCAST recommendations

**DOI:** 10.1186/s12879-019-4721-9

**Published:** 2019-12-30

**Authors:** Kathrin Rothe, Nina Wantia, Christoph D. Spinner, Jochen Schneider, Tobias Lahmer, Birgit Waschulzik, Roland M. Schmid, Dirk H. Busch, Juri Katchanov

**Affiliations:** 1 Technical University of Munich, School of Medicine, Institute for Medical Microbiology, Immunology and Hygiene, Trogerstr. 30, 81675 Munich, Germany; 2 German Centre for Infection Research (DZIF), Partner Site Munich, Munich, Germany; 3 Department of Medicine II, Technical University of Munich, School of Medicine, University Hospital rechts der Isar, Munich, Germany; 4 Institute of Medical Informatics, Statistics, and Epidemiology, Technical University of Munich, Munich, Germany

**Keywords:** Community-onset bacteraemia, Emergency department, Blood cultures, EUCAST, Antimicrobial susceptibility pattern, Treatment regimen

## Abstract

**Background:**

This study investigated predominant microorganisms causing community-onset bacteraemia at the medical emergency department (ED) of a tertiary-care university hospital in Germany from 2013 to 2018 and their antimicrobial susceptibility patterns.

**Methods:**

Antimicrobial resistance patterns in patients with positive blood cultures presenting to an internal medicine ED were retrospectively analysed.

**Results:**

Blood cultures were obtained at 5191 of 66,879 ED encounters, with 1013 (19.5%) positive results, and true positive results at 740 encounters (diagnostic yield, 14.3%). The most frequently isolated relevant microorganisms were *Enterobacterales* (*n* = 439, 59.3%), *Staphylococcus aureus* (*n* = 92, 12.4%), *Streptococcus pneumoniae* (*n* = 34, 4.6%), *Pseudomonas aeruginosa* (*n* = 32, 4.3%), *Streptococcus pyogenes* (*n* = 16, 2.2%), *Enterococcus faecalis* (*n* = 18, 2.4%), and *Enterococcus faecium* (*n* = 12, 1.6%). Antimicrobial susceptibility testing revealed a high proportion of resistance against ampicillin-sulbactam in *Enterobacterales* (42.2%). The rate of methicillin-resistant *Staphylococcus aureus* was low (0.4%).

Piperacillin-tazobactam therapy provided coverage for 83.2% of all relevant pathogens using conventional breakpoints. Application of the new European Committee on Antimicrobial Susceptibility Testing (EUCAST) recommendations increased the percentage of susceptible isolates to high-dose piperacillin-tazobactam to 92.8% (*p* < 0.001). Broad-spectrum carbapenems would only cover an additional 4.8%. The addition of vancomycin or linezolid extended coverage by just 1.7%.

**Conclusions:**

Using an ureidopenicillin-beta-lactamase inhibitor combination at the high dose suggested by the new EUCAST recommendations provided nearly 93% coverage for relevant pathogens in patients with suspected bloodstream infection in our cohort. This might offer a safe option to reduce the empiric use of carbapenems. Our data support the absence of a general need for glycopeptides or oxazolidinones in empiric treatment.

## Background

In the United States, bacteraemia affects approximately 200,000 patients per year, with 10 per 1000 cases requiring hospital admission [1]. The incidence of community-acquired bloodstream infections is increasing [2]. Previous studies in Denmark and Canada, for example, reported incidence rates around 100 episodes per 100,000 per year [3, 4]. In Europe, there are only a few population-based surveillance systems [5, 6]. Community-onset bacteraemia is a common problem in patients visiting emergency departments (EDs) [7, 8], and bacteraemia is associated with significant healthcare costs and mortality. The bacteraemia-associated mortality rate has been shown to decrease with early administration of appropriate antimicrobial therapy [9–13]. Thus, ED physicians must regularly initiate empirical antimicrobial therapy before the results of blood cultures (BCs) are available.

BCs are the gold standard and the most important first-line tool for diagnosing bacteraemia and severe bacterial infections, including sepsis [14–17]. Diagnostic bundles, especially blood culture diagnostics in the ED setting, have been shown to increase numbers of sepsis diagnoses [18], and educational interventions have been shown to improve outcomes in patients with sepsis [19]. Sepsis detection is vital for optimal patient care in the ED setting, as sepsis mortality is reduced when correct antimicrobial therapy is initiated promptly [20–22]. According to national and international sepsis guidelines [23, 24], empiric broad-spectrum antimicrobial therapy with coverage of the most likely pathogens must be initiated immediately in patients with sepsis, including those with bacteraemia [20, 21]. Additionally, it is an important concept in sepsis care to de-escalate antimicrobial therapy once pathogen identification and resistance testing are completed, or to discontinue empiric antimicrobial therapy when infection can be ruled out [23].

When initiating empiric antibiotic therapy, the potential infectious focus, emergence of multi-resistant pathogens, and economic efficiency must be considered [25]. Common regimens of empiric antimicrobial therapy in the ED are aminopenicillin-beta-lactamase inhibitor combinations (such as ampicillin-sulbactam), ureidopenicillin-beta-lactamase inhibitor combinations (such as piperacillin-tazobactam), second and third generation cephalosporins (such as cefuroxime and ceftriaxone), and carbapenems (such as imipenem and meropenem). Hence, knowledge of the expected spectrum of pathogens and antimicrobial resistance is of paramount importance.

The German national 2015 GERMAP report [26] showed that over 10% of *Escherichia coli* strains from hospital care in Germany were extended-spectrum beta-lactamase positive, with a piperacillin-tazobactam resistance rate of 6.2%. Hospital strains of *Klebsiella oxytoca* and *Enterobacter cloacae* showed piperacillin-tazobactam resistance rates as high as 20.8 and 33.5%, respectively.

However, data on expected pathogens and antimicrobial susceptibility, particularly in community-acquired bacteraemia, are remarkably sparse in Europe [27–32]. Guidelines on antibiotic stewardship recommend adapting guidelines for empiric therapy to local microbiological data and resistance rates to reduce the use of reserve antibiotics. The empiric therapy must be effective, cover the most common pathogens and its prompt initiation is associated with more favourable outcomes [33]. However, to avoid the emergence of antimicrobial resistance, the narrowest spectrum agents and de-escalation strategies are crucial [34]. In addition, in January 2019, the European Committee on Antimicrobial Susceptibility Testing (EUCAST) modified the definitions to categorise susceptibility testing of microorganisms, resulting in a new classification of bacteria as susceptible-standard exposure, susceptible-increased exposure (formerly intermediate), and resistant [35]. This new classification is now related to adequate dosing and exposure at the site of infection. Therefore, ED physicians must be aware of recommended dosing strategies, which have to be addressed at educational visits by the clinical microbiologist or the antibiotic stewardship team.

We conducted this study to expand existing knowledge on expected pathogens and antimicrobial susceptibility in community-acquired bacteraemia in Europe, as well as to explicitly analyse whether the very recent major alterations in EUCAST susceptibility testing classification have implications for empirical antimicrobial therapy.

Thus, we aimed to investigate the predominant microorganisms causing community-onset bacteraemia at the medical ED of a tertiary care university hospital in Germany and their antimicrobial susceptibility patterns, in consideration of the new EUCAST recommendations.

## Methods

We performed a retrospective analysis of all BCs collected at the internal medicine ED of a university hospital with approximately 1200 beds, located at the centre of Munich, Germany, between 1 October 2013 and 31 September 2018, using the HyBase® analysis system (epiNet AG, Bochum, Germany). The medical ED is responsible for all non-surgical emergencies.

Major indications for ED physicians to suspect infection, obtain BCs [36], and consider antimicrobial therapy include the following: clinically suspected organ infection with accompanying bacteraemia (such as meningitis, cholecystitis, pyelonephritis, necrotising fasciitis, osteomyelitis, severe pneumonia, endocarditis, vascular graft infection, or prosthetic joint infection), clinically suspected sepsis, defined by published sepsis scores, such as the sepsis-related organ failure assessment score, quickSOFA (qSOFA) [22] or SIRS criteria [37], or detected or reported body core temperature ≥ 38.3 °C.

Elevated white blood cell count, C-reactive protein, and procalcitonin, as well as patient’s clinical status, were also taken into consideration for obtaining BCs and empiric initiation of antimicrobial therapy. The actual decision to start antibiotic therapy was at the discretion of the physician in charge.

Blood was obtained at the bedside exclusively by physicians according to national practice after skin decontamination using the local antiseptic Octeniderm® (Schülke & Mayr GmbH, Norderstedt, Germany). Blood was inoculated into aerobic and anaerobic BC media (BACTEC™ Plus, Becton Dickinson, Sparks, MD, United States of America) suitable for processing via an automated BC system (BACTEC™ Fluorescent Series, Becton Dickinson). The culture bottles were incubated for 5–7 days according to the manufacturer’s recommendations. Immediate Gram stain identification, species identification (Matrix Assisted Laser Desorption Ionization-Time of Flight Mass Spectrometry, Bruker Daltronics, Leipzig, Germany) and automated antimicrobial susceptibility testing (VITEK®, bioMerieux, Marcy l’Etoile, France) were performed for all positive cultures. Anaerobic strains were tested using minimal inhibitory concentration (MIC) test strips (Liofilchem Inc., Waltham, MA, United States of America).

The majority of BC specimens were obtained by peripheral venepuncture and directly from indwelling catheters when catheter-related bacteraemia was suspected. The yield of true bacteraemia was defined as the percentage of episodes with positive BC results, yielding true positive pathogens (not contaminants such as coagulase-negative staphylococci [CoNS] from a single culture alone) for all episodes during which BCs were obtained. All isolates were categorised as true-positives or contaminants by dichotomous categorisation after critical assessment by at least two investigators, including a clinical microbiologist and a clinical care physician.

Considering the difficulty in determining the clinical significance of CoNS, these isolates were reviewed separately on the basis of the number of positive culture sets, the presence of intravascular devices or indwelling catheters with catheter-related bloodstream infection (CLABSI), the presence of prosthetic heart valves, and patients’ characteristics [9]. The isolates were considered clinically significant if two or more bottles out of two or three BC sets yielded the same CoNS [9]. Prosthetic valve endocarditis was extremely scarce in the study population. Hence, the detection of CoNS was usually benign, and often represented contamination. Therefore, we excluded CoNS from considerations for empiric antimicrobial therapy and the term relevant positive BCs is defined as true positive BCs excluding true CoNS -bacteraemia.

Additionally, assessment for bacteraemia with viridans group streptococci [38–40] was carried out on the basis of type (e.g., ‘abscess-forming’ *Streptococcus anginosus* group versus ‘low-virulence’ *Streptococcus mitis* group), focus of infection, and patients’ risk factors (such as relevant systemic immunosuppression) to discriminate true positive bacteraemia from contamination. The focus of infection was defined as the clinically most likely site of infection responsible for bacteraemia based on the analysis of clinical signs, microbiological findings, and imaging results [9, 41].

EUCAST has very recently modified the definitions for categorisation of susceptibility testing of microorganisms, resulting in a new classification system of bacteria as susceptible-standard exposure, susceptible-increased exposure, and resistant. EUCAST clinical breakpoints 2012–2019 (breakpoint tables version 2.0–version 9.0) for *Enterobacterales* and *Pseudomonas aeruginosa* were therefore revised regarding variability in MIC breakpoints for ampicillin-sulbactam, piperacillin-tazobactam, imipenem, meropenem, cefuroxime, ceftriaxone, ceftazidime, ciprofloxacin, and moxifloxacin. When the MIC breakpoint values did not at all differ during the entire period, the new EUCAST intermediate definition (susceptible-increased exposure) was hypothetically retrospectively applied, leading to an increase in treatable (susceptible-increased exposure) isolates for ampicillin-sulbactam, piperacillin-tazobactam, meropenem, cefuroxime, ceftriaxone, and ceftazidime.

Statistical analyses were performed using IBM SPSS Statistics V.25.0 (IBM Corp, Armonk, NY). To test the consistency in responses of susceptibility-testing across the two variables ‘novel EUCAST classification’ and ‘conventional breakpoints’ we used the McNemar test (two-sided, significance level of 0.05). Exact 95% confidence intervals were calculated for the percentages of covered pathogens (using the online calculator https://www.causascientia.org/math_stat/ProportionCI.html).

## Results

During the study period, 66,879 patient encounters within the ED were recorded. Female and male patients were equally represented in the ED (49.8 and 50.2%, respectively). The mean age was 54.2 (±20.8) years.

Presentations through the ambulance and emergency medical services accounted for 35% of all ED encounters, while walk-in patients accounted for the remaining 65%. BCs were obtained at 5191 encounters (7.8%); 37,850 encounters resulted in admission to the hospital (56.6%), with approximately 2.5% of patients admitted directly to intensive care units.

True bacteraemia was detected in 740 ED cases, resulting in a BC diagnostic yield of 14.3% (740 out of 5191). The rate of community-onset true bacteraemia among patients presenting to the ED was 1.1%, and 42 bacteraemia episodes were polymicrobial (5.6% of true bacteraemia episodes).

Pathogens considered as contaminants were present in 4.1% of all positive BCs. Contaminants were the only cause of bacteraemia in 266 episodes (5.1%) of all ED encounters in which BCs were obtained and 26.3% of all encounters in which bacteraemia was detected. As the detection of CoNS was usually benign, and often represented contamination without a need for initial empiric antimicrobial therapy, they were excluded from further considerations for schemas regarding empiric antimicrobial therapy.

Consistent with previously published data [28], *Enterobacterales* were the most commonly isolated pathogens in cases of true bacteraemia (*n* = 439, 59.3%) followed by *Staphylococcus aureus* (*n* = 92, 12.4%). *Streptococcus* species accounted for 13.5% of true bacteraemia with *Streptococcus pneumoniae* (*n* = 19) being the most frequently isolated, followed by *Streptococcus pyogenes* (*n* = 16). *Pseudomonas aeruginosa* (*n* = 32) and *Enterococcus* species (*n* = 30) bacteraemia represented 4.3 and 4.0% of all true bacteraemia cases, respectively.

Anaerobes accounted only for a small fraction of community-onset bacteraemia cases in our ED cohort: 12 bacteraemia episodes with *Bacteroides* species (*Bacteroides fragilis* was detected in 10 episodes) and 12 bacteraemia episodes with *Clostridium* species (7 of these being *Clostridium perfringens*) were observed. All except one anaerobic isolate were susceptible to ampicillin-sulbactam: only one detected *Bacteroides fragilis* isolate was ampicillin-sulbactam-resistant.

After exclusion of CLABSI with CoNS, which do not obligatorily have to be covered by empirical treatment in the ED as described above, bloodstream infections (BSI) with *Enterobacterales*, *Staphylococcus aureus*, *Streptococcus* species*, Pseudomonas aeruginosa*, and *Enterococcus* species accounted for more than 95% of all episodes of relevant positive BCs in the ED. Table 1 summarises the results of antimicrobial susceptibility testing for those pathogens most commonly isolated in the present study.

**Table 1:** Antimicrobial susceptibility data for the main pathogens in BSI in the ED

Microorganisms (number of isolates, percentage of all relevant positive BCs), *n* = 643	*Staphylococcus aureus* (*n* = 92, 12.4%)	*Streptococcus pyogenes* (*n* = 16, 2.2%)	*Streptococcus pneumoniae* (*n* = 34, 4.6%)	*Enterococcus faecalis* (*n* = 18, 2.4%)	*Enterococcus faecium* (*n* = 12, 1.6%)	*Enterobacterales* (*n* = 439, 59.3%)	*Pseudomonas aeruginosa* (*n* = 32, 4.3%)
Penicillin	N/A	16 (100%)	33 (97.1%)	–	–	–	–
Ampicillin	N/A	16 (100%)	33 (97.1%)	18 (100%)	3 (25%)	155 (35.3%)	–
Flucloxacillin	89 (96.7%)	N/A	–	–	–	–	–
Ampicillin-sulbactam	89 (96.7%)	16 (100%)	33 (97.1%)	18 (100%)	3 (25%)	230 (52.4%) 253* (57.8%)	–
Piperacillin-tazobactam	89 (96.7%)	16 (100%)	33 (97.1%)	18 (100%)	3 (25%)	376 (85.6%) 411* (93.8%)	25 (78.1%) 27 (84.4%)
Imipenem	89 (96.7%)	16 (100%)	34 (100%)	18 (100%)	3 (25%)	426 (97.0%)	26 (81.3%)
Meropenem	89 (96.7%)	16 (100%)	34 (100%)	–	–	439 (100%) 439* (100%)	26 (81.3%) 29* (90.6%)
2nd gen Cephalosporin (cefuroxime)	89 (96.7%)	16 (100%)	33 (97.1%)	–	–	327 (74.5%) 347* (79.0%)	–
3rd gen Cephalosporin (ceftriaxone)	89 (96.7%)	16 (100%)	34 (100%)	–	–	388 (88.4%) 389* (88.6%)	–
4th gen Cephalosporin (ceftazidime)	–	–	–	–	–	385 (87.7%) 412* (93.8%)	28 (87.5%) 29* (90.6%)
Ciprofloxacin	86 (93.5%)	–	–	N/A	N/A	343 (78.1%)	26 (81.3%)
Levofloxacin	86 (93.5%)	15 (93.8%)	34 (100%)	N/A	N/A	N/A	N/A
Moxifloxacin	86 (93.5%)	N/A	N/A	–	–	322 (73.3%)	–
Vancomycin	92 (100%)	16 (100%)	34 (100%)	18 (100%)	12 (100%)	–	–
Linezolid	92 (100%)	16 (100%)	34 (100%)	18 (100%)	12 (100%)	–	–

For *Enterobacterales* and *Pseudomonas aeruginosa*, the percentage of susceptible isolates is presented according to the interpretation of breakpoints at the time of testing. According to the novel EUCAST definition of the intermediate category, therapeutic success is likely if exposure is increased. This was hypothetically retrospectively applied, leading to an increase in susceptible isolates if dosing recommendations were obeyed (marked with *). “-” indicates that the species is a poor target for therapy with the agent; isolates may be considered as resistant. N/A indicates not available/applicable; relevant positive BCs are defined as true positive BCs excluding true CoNS -bacteraemia.

The new category ‘susceptible-increased exposure’ within the novel EUCAST classification broadens the susceptible zone if adequate dosing and exposure at the site of infection are guaranteed. For *Enterobacterales* and *Pseudomonas aeruginosa*, hypothetical retrospective application of the new EUCAST definitions resulted in a significant increase in isolates treatable by ampicillin-sulbactam (*p* < 0.001), piperacillin-tazobactam (*p* < 0.001) and cefuroxime (p < 0.001) if dosing recommendations were obeyed. Assuming that all isolates of *Enterobacterales* (*n* = 439) and *Pseudomonas aeruginosa* (*n* = 32) in bacteraemia formerly classified as ‘intermediate’ (*n* = 37) would have been treated with carbapenems and could now be treated with piperacillin-tazobactam, this results in a hypothetical reduction of carbapenem use of 7.9% for *Enterobacterales* and *Pseudomonas aeruginosa*.

Common regimens of empiric antimicrobial therapy in the ED such as ampicillin-sulbactam, piperacillin-tazobactam, cephalosporins, and carbapenems were analysed regarding enclosure of the microorganism detected as causing bacteraemia in the study population. Table 2 summarises the percentage of BSI that would be covered by these different empiric antimicrobial regimens in the ED population.

**Table 2:** Percentages of pathogens covered in bacteraemia presenting to the ED by different empiric antimicrobial regimens

Empiric antimicrobial treatment selection	% of covered pathogens(95%CI)	% of covered pathogens, new EUCAST recommendations (95%CI)	*p*-Value	Bacteria without sufficient coverage
Aminopenicillin-beta-lactamase inhibitor combinations (ampicillin-sulbactam)	60.5% (56.8%;64.4%)	63.9% (60.4%;67.8%)	*p* < 0.001	ESBL*-Enterobacterales*, *Pseudomonas aeruginosa*, *Enterococcus faecium*, MRSA
Ureidopenicillin-beta-lactamase inhibitor combinations (piperacillin-tazobactam)	83.2% (80.1%;85.9%)	92.8% (90.6%;94.6%)	*p* < 0.001	*Enterococcus faecium* MRSA
2nd generation cephalosporins (cefuroxime)	72.3% (69.7%;76.5%)	75.4% (72.9%;79.5%)	*p* < 0.001	ESBL*- Enterobacterales*, *Pseudomonas aeruginosa*, *Enterococcus spp.*, MRSA
3rd generation cephalosporins (ceftriaxone)	82.0% (79%;84.9%)	82.1% (79%;84.9%)	*p* = 1.000	ESBL- *Enterobacterales*, *Pseudomonas aeruginosa*, *Enterococcus spp.*, MRSA
Carbapenems (imipenem or meropenem)	97.0% (95.6%;98.2%)	97.5% (96.2%;98.6%)	*p* = 0,250	*Enterococcus faecium* MRSA
COMBINATION REGIMENS				
Piperacillin-tazobactam + vancomycin	85.0% (81.9%;87.5%)	94.6% (92.5%;96.1%)	*p* < 0.001	
carbapenems (meropenem/ imipenem) + vancomycin	98.8% (97.8%;99.5%)	99.3% (98.4%;99.7%)	*p* = 0,250	
Piperacillin-tazobactam + linezolid	85.0% (82.1%;87.6%)	94.6% (92.7%;96.2%)	*p* < 0.001	
Carbapenem (meropenem/ imipenem) + linezolid	98.8% (98%;99.6%)	99.3% (98.6%;99.8%)	*p* = 0,250	

Table based on the data of most frequent bacteraemia infections in the study population, 2013–2018. CoNS were excluded from the analysis as they represented mostly contamination or CLABSI with special requirements for therapy. McNemar test (two-sided, significance level 0.05) was used to compare results between novel EUCAST classification and conventional breakpoints. Exact 95% confidence intervals are presented for the percentages of covered pathogens. Abbreviations: *ESBL* extended-spectrum beta-lactamase, *MRSA* methicillin-resistant *Staphylococcus aureus*, *CoNS* coagulase-negative staphylococci, 95%CI: 95% Confidence Interval.

### Specific bacteraemia cases

BSI with *Pseudomonas aeruginosa* was detected at 32 ED encounters. All affected patients had pre-existing medical conditions and frequently had prior contact with the health care system. Advanced cancer was present in 24 of these patients (75%) with hematologic malignancy accounting for half of these cases. Neutropenia was present in 12 patients and status post solid organ transplantation in 4 patients with *Pseudomonas aeruginosa* bacteraemia.

BSI with *Enterococcus faecium* was rare in patients presenting to the ED; only 12 cases were detected over the 5-year study period. Six patients suffered from acute obstructive cholangitis, two patients from peritoneal dialysis-associated peritonitis, and five patients from advanced cancer.

## Discussion

In ED patients with suspected BSI, antimicrobial therapy has to be initiated before the causative microorganism can be identified and often also before the focus of the infection is unequivocally identified [25]. BC remains the gold standard and first line tool for diagnosing severe bacterial (bloodstream) infections [14, 15]. The detection of relevant microorganisms in the BC facilitates the search for an infectious focus and allows adjustment to a directed antimicrobial therapy. Only prompt pathogen identification and susceptibility testing allows de-escalation of empiric antimicrobial therapy [34] and widens existing knowledge of the bacterial spectrum and antimicrobial resistance in BSI; therefore, BC represents a major pillar of antibiotic stewardship [42]. It is of paramount importance for ED physicians to be aware of the expected spectrum of pathogens in bacteraemia and antimicrobial susceptibilities. In accordance with previous studies, our data show that *Enterobacterales*, *Staphylococcus aureus*, *Streptococcus* species, and *Pseudomonas aeruginosa* account for the majority of clinically relevant BSI in the ED [9, 43–45].

In our study population, empirical therapy with ureidopenicillin-tazobactam covered 83.2% of all relevant pathogens. However, this proportion increased to 92.8% after including the isolates that were hypothetically retrospectively classified under the category of susceptible-increased exposure according to the novel EUCAST definitions. Applying the novel EUCAST breakpoints and using the high-dose antimicrobial regimens would therefore provide an option of broad microbiological efficacy in the vast majority of patients presenting with community-onset bacteraemia, while reducing carbapenem use. However, in this potential antibiotic stewardship intervention approach, awareness of high-dose recommendations is crucial. The education of physicians, nurses, and other staff might be essential for the success of such a strategy. Using a broad spectrum carbapenem would only cover an additional 4.8% of relevant pathogens (28 isolates of *Enterobacterales* and three isolates of *Pseudomonas aeruginosa* in the study population). In the study population, of 35 isolates of *Enterobacterales* that were retrospectively hypothetically classified under the category of susceptible-increased exposure, 19 (54.3%) tested positive for extended-spectrum beta-lactamase (ESBL).

Treatment of community-onset BSI due to ESBL-producing *Enterobacterales* with an ureidopenicillin-beta-lactamase inhibitor combination is still a matter of debate and conflicting results have been reported. It has been shown that piperacillin-tazobactam is not inferior to carbapenems for treatment of extended-spectrum beta-lactamase (ESBL)-producing *Enterobacterales* especially in low-to moderate-severity infections and when adequate dosing regimens (4.5 g every 6 h or 4.5 g every 8 h as prolonged infusion) are used [46–50]. Tamma et al. [51] reported that carbapenem therapy is associated with improved survival compared to piperacillin-tazobactam in patients with ESBL-bacteraemia. This study used piperacillin-tazobactam 3.375 g every 6 h in the majority of patients. Notably, when high- dose piperacillin-tazobactam (defined as 4.5 g every 6 h) was administered to a subset of patients in this study, no difference in clinical outcome compared to carbapenem administration was observed [51]. The lack of uniform treatment regimens regarding piperacillin-tazobactam-dosing has to be taken into consideration when comparing outcomes of different studies on this topic.

Of note, a study conducted by Harris et al. [49], showing non-inferior 30-day mortality of piperacillin-tazobactam-therapy in ESBL-bacteraemia, explicitly administered piperacillin-tazobactam 4.5 g every 6 h. In contrast to other studies, in this case susceptibility testing was performed according to EUCAST standards. Remarkably the EUCAST breakpoint for susceptibility testing for piperacillin-tazobactam (susceptible standard exposure: MIC ≤8 mg/L) is set lower than the Clinical and Laboratory Standards Institute (CLSI) breakpoint (susceptible: MIC ≤16 mg/L) [49]. The inconsistent breakpoints of EUCAST and CLSI can result in different interpretation of susceptibility testing, impeding comparability of studies even more. Therefore, additional research is needed to provide data on the question if piperacillin-tazobactam is a safe therapeutic option for critical ill patients with severe infections with ESBL-producing *Enterobacterales*, especially considering the source of infection, dosing regimens, results of susceptibility testing and MICs [50].

It has been shown, that community onset-bacteraemia with ESBL-producing *Escherichia coli, Klebsiella* species and *Proteus mirabilis* is associated with worse clinical outcome compared to non ESBL-producing isolates, but a propensity-matched analysis did not show a significant difference between appropriateness of empirical antibiotic therapy between the groups [52]. Remarkably in the busy ED setting, primarily identification of ESBL-bacteraemia by adequate blood culture diagnostic is vital, thus enabling subsequent adjustment of antimicrobial therapy in this population at risk. However, in clinically stable ED-patients in absence of septic condition and low prevalence of ESBL-producers, it seems reasonable to start with empiric antimicrobial therapy according to local resistance rates [53]. Definite antimicrobial therapy should then be adopted to results of pathogen identification, resistance testing and clinical course.

In conclusion, strategies to reduce the use of carbapenems are warranted and safe carbapenem-sparing treatment options with ureidopenicillin-beta-lactamase inhibitor combinations might be one possible approach. Therefore, we propose the option of adequate high-dose ureidopenicillin-beta-lactamase inhibitor combination therapy for community-onset bacteraemia with the intent to reduce carbapenem overtreatment especially in low-to moderate-severity infections. Nevertheless, awareness of risk factors that accompany the need for distinct antimicrobial coverage and specific therapeutic considerations necessary in critically ill patients (who require intensive care) must be raised.

One particular target population for broad spectrum antibiotic prescription is patients with sepsis. Many patients with bacteraemia fulfil sepsis criteria [54–57], but only a fraction of patients with sepsis have detectable bacteraemia [58–61]. Our study investigated the ED setting of a German tertiary care hospital. The ED physician must recognise suspected sepsis in a timely manner and promptly begin the initial work-up, including obtaining blood cultures and administering broad spectrum antibiotics. The patient’s clinical condition is the determining factor for deciding the necessity of initiating antimicrobial therapy and selecting suitable antimicrobial agents. The urgent need to immediately initiate the correct antibiotic in patients with sepsis creates the need for broad-spectrum agents [62]. Our study shows that piperacillin-tazobactam monotherapy at the high dose suggested in the new EUCAST recommendations covers nearly 93% of bacteraemia cases in our cohort. This offers a safe option for suspected BSI in the ED and reduces empiric use of carbapenems in line with antibiotic stewardship principles. In particular, in clinically stable patients with suspected BSI who require immediate initiation of empiric broad-spectrum antibiotics, it seems reasonable to start with a narrower spectrum therapy according to local resistance rates and withhold reserve antibiotics when clinical monitoring for deterioration is provided.

Caution is warranted in the care of patients at risk of multi-resistant pathogens, such as patients with immunosuppression or haematological malignancy. General risk factors for multi-resistant bacteria include previous colonisation, previous hospitalisation, previous antibiotic therapy, patients in long-term-care facilities requiring chronic care or with chronic wounds, and patients with permanent catheters [63–65]. Adding vancomycin or linezolid widened coverage by only an additional 1.7% in the present study and should therefore be restricted, especially to patients with clinical suspicion for CLABSI or BSI due to methicillin-resistant *Staphylococcus aureus.* It is not generally needed in routine empiric therapy. In the study population, the rate of methicillin-resistant *Staphylococcus aureus* (MRSA) in community-acquired BSI was extremely low. Generally, the percentage of invasive *Staphylococcus aureus* isolates with resistance to methicillin in Germany is decreasing, with a rate of 9.1% in 2017 [66]. Surveillance systems in Germany show a decline in incidence of MRSA infections to 4.8/100,000 in 2014 with regional differences; incidences were as low as 2.0/100,000 in 2014 in southern Germany [67]. We therefore believe that, in absence of known previous risk factors, it is possible to withhold vancomycin or linezolid from most patients with suspected BSI in our ED setting.

The present study supports the fact that in patients with suspected BSI the use of reserve antibiotics such as carbapenems, vancomycin and oxazolidinones is only essential in very few cases (6.5%) with dedicated risk factors within special populations. Prina and colleagues proposed the acronym ‘PES’ (‘*Pseudomonas aeruginosa*, Enterobacteriaceae extended-spectrum beta-lactamase-positive and methicillin-resistant *Staphylococcus aureus’*) to identify pathogens that require different treatment regimes in community-acquired pneumonia [68]. For BSI in the ED, *E. faecium* should be considered as a microorganism usually requiring special antimicrobial treatment. However, the number of patients with community-onset *E. faecium* bacteraemia was very low, and half of the episodes presented as acute cholangitis, where source control was the main pillar of therapy. Moreover, MRSA- bacteraemia was extremely rare, with only three cases observed in the study period. Nevertheless, awareness of the risk factors that accompany the need for distinct antimicrobial coverage must be raised and using an acronym might be one possible method to address this issue. Additional research is needed to provide guidance for the treatment of critically ill patients with septicaemia that require intensive care, with the aim of increasing coverage of empirical antibiotic therapy to 100%.

## Limitations

This study has several limitations. It is a single-centre study based on a retrospective analysis of laboratory data without correlation with clinical outcome data. However, the heterogeneous population of a city-based ED with a large catchment area may allow generalisation to other EDs in Germany and Europe. Another limitation of the present study is that only bacteraemia was considered. The sensitivity of BC is limited; moreover, some pathogens accounting for acute infections in the ED, such as *Legionella pneumophila* or fastidious intracellular pathogens, cannot be isolated using BC techniques. These pathogens must be taken into consideration and clinical indicators must be carefully observed. It is also important to differentiate between the clinical condition of sepsis and the microbiological finding of bacteraemia. Bacteraemia might occur during sepsis, but also affects clinically stable patients without sepsis. Although bacteraemia might strongly correlate with suspected sepsis, the findings of the present study cannot lead to a clinical recommendation for sepsis patients in general, as this study was focused on ED encounters with suspected BSI and antimicrobial resistance of bacteraemia; no clinical data on sepsis diagnosis, antimicrobial treatment, or therapeutic outcome was available.

## Conclusions

BCs remain the gold standard for diagnosing severe bacterial infections [14, 15], representing a major pillar of infection diagnosis and a crucial part of antibiotic stewardship [42]. Our study indicates that, at a tertiary care centre in Germany, empiric therapy with a high-dose ureidopenicillin-beta-lactamase inhibitor combination, in accordance with the novel EUCAST recommendations, might be an appropriate antibiotic stewardship intervention strategy for reducing carbapenem use in patients with suspected community-onset bacteraemia. In our cohort, more than 90% of detected relevant microorganisms in BSI were successfully covered with this antibiotic regimen. Still, caution is warranted in critically ill patients who require intensive care, patients with risk factors for infections with multi-resistant pathogens [63–65], and/or patients with vascular-device-related BSI, as our data does not address these populations. Here, specific therapeutic considerations are necessary. Moreover, the therapeutic role of high-dose ureidopenicillin-beta-lactamase inhibitor combinations in infections due to extended-spectrum beta-lactamase *Enterobacterales* must be further clarified in clinical studies. Incorporating the new EUCAST definitions in antibiotic stewardship programmes could enhance carbapenem withholding and restrict the use of reserve antibiotics to fight emerging resistance.

## Data Availability

All relevant data have been made available in the article. Raw data can be requested from the corresponding author.

## Abbreviations

95%CI: 95% Confidence Interval
BC: Blood culture
BSI: Bloodstream infection
CLABSI: Catheter-related bloodstream infection
CoNS: Coagulase-negative staphylococci
ED: Emergency department
ESBL: Extended-Spectrum Beta-Lactamase
EUCAST: European Committee on Antimicrobial Susceptibility Testing
MRSA: Methicillin-resistant *Staphylococcus aureus*
